# Impact of Box-Cox Transformation on Machine-Learning Algorithms

**DOI:** 10.3389/frai.2022.877569

**Published:** 2022-04-07

**Authors:** Luca Blum, Mohamed Elgendi, Carlo Menon

**Affiliations:** Biomedical and Mobile Health Technology Laboratory, ETH Zurich, Zurich, Switzerland

**Keywords:** Box-Cox transformation, power transformation, Non-linear mappings, feature transformation, accuracy improvement, classifier optimization, preprocessing data, monotonic transformation

## Abstract

This paper studied the effects of applying the Box-Cox transformation for classification tasks. Different optimization strategies were evaluated, and the results were promising on four synthetic datasets and two real-world datasets. A consistent improvement in accuracy was demonstrated using a grid exploration with cross-validation. In conclusion, applying the Box-Cox transformation could drastically improve the performance by up to a 12% accuracy increase. Moreover, the Box-Cox parameter choice was dependent on the data and the used classifier.

## Introduction

Feature transformation can improve the performance of a machine learning algorithm. Simple transformations already had a significant impact on classification performance (Bicego and Baldo, 2016; Liang et al., 2020). Motivated by their findings, the impact of the Box-Cox transformation for classification tasks was studied. Often, Box-Cox is used to increase the Gaussianity of data. This can help in some special cases; however, we observed that transformations that do not maximize the Gaussianity of the data are often superior for classification accuracy. Additionally, Bicego and Baldo (2016) have shown that the Gaussianity of datasets is not critical and by allowing the effect of the Box–Cox transformation work in operational ranges that do not necessarily correspond to an increase in Gaussianity, they have shown that class separability can be improved. Furthermore, they proposed an automatic procedure for obtaining an optimal transformation. Their procedure relied on the *spherical* and *diagonal* optimization of statistical measurements, such as maximum likelihood or Fisher criterion. They showed that both are capable of improving the classification result, although the *diagonal* case often gives higher accuracy. This can be expected due to the higher number of parameters. Furthermore, they demonstrated that the choice of optimization criteria depends on the classifier itself.

Gao et al. (2017) attempted to find the optimal Box-Cox transformation in big data. They focused on regression and tried to get a maximum likelihood estimation (MLE) for the Box-Cox parameter when the dataset is massive. By using MapReduce, they proposed an algorithm that can be run in parallel and is able to process big data in chunks.

Cheddad (2020) investigated the effect of the Box-Cox transformation on images. They proposed an image pre-processing tool by using the Box-Cox transformation for histogram transformation. The parameters for the transformation were calculated using the MLE. By using image histograms instead of the image data, the time complexity could be kept static, and thus independent of the size of the image.

However, our focus is on the classification of tabular data that fits into the main memory. We sought to explore a generalization of the approach from Bicego and Baldo (2016) and provide an optimization procedure that is classifier dependent.

## Box-Cox Transformation

The original Box-Cox transformation is a one-dimensional transformation with one parameter often called λ and is applied element-wise to a vector *y* (Box and Cox, 1964):


$$\begin{array}{l} \text{Let }y\in {\mathbb{R}}^{n}\text{ and }\lambda \in \mathbb{R}\\ \;y_{i}^{\left(\lambda \right)}=\{\begin{array}{ll} \frac{y_{i}^{\left(\lambda \right)}-1}{\lambda } & \text{if }\lambda \neq \text{0}\\ \ln\left(y_{i}\right) & \text{if }\lambda \text{=0} \end{array} \end{array}$$

Many different criteria have been proposed for an optimal λ. The most used method, which was introduced by Box and Cox (1964), is a MLE. Other approaches include a Bayesian approach (Sweeting, 1984), robust estimators, Carroll and Ruppert (1985), Lawrance (1988), and Kim et al. (1996) and an attempt to iteratively maximize Gaussianity (Vélez et al., 2015). The Box-Cox transformation is mostly studied for regression tasks. For λ>1 the transformation is convex and for λ <1 the transformation is concave. As described by Bicego and Baldo (2016), the data is stretched in the positive direction for λ>1 and stretched in the negative direction for λ <1. Assuming the data is range standardized between 1 and 2, this means for λ>1 that data points near 1 have a smaller relative distance than points near 2 after applying the Box-Cox transformation (Bicego and Baldo, 2016). The opposite behavior holds for λ <1 (Bicego and Baldo, 2016). For λ = 1 the data is only shifted by 1 in the negative direction. The Box-Cox transformation is monotonic and therefore does not change the ordering of the data. These properties might help to increase class separability. For multi-dimensional data, *X*∈ℝ^*n*×*p*^, it is usually applied *p* times as 1-dimensional mapping to each column with different values for λ. Therefore, the overall transformation is specified by a *p*-dimensional vector, Λ = [λ_1_, λ_2_, …, λ_*p*_].

The optimization of the parameter vector Λ can be done in several ways. Naturally, one could optimize λ_*i*_ of the corresponding column *X*_*i*_ independently with traditional criteria such as MLE (Box and Cox, 1964) or the Bayesian approach (Sweeting, 1984). This will be referred to as *diagonal* setting,


(1) $$\begin{array}{r} \Lambda^{*}=\left[\begin{array}{c} \lambda_{1}^{*}\\ \lambda_{2}^{*}\\ ...\\ \lambda_{p}^{*} \end{array}\right]=\left[\begin{array}{c} \underset{\lambda_{1}}{\mathrm{argmin}}L\left(\lambda_{1},\;X_{1}\right)\\ \underset{\lambda_{2}}{\mathrm{argmin}}L\left(\lambda_{2},\;X_{2}\right)\\ ...\\ \underset{\lambda_{p}}{\mathrm{argmin}}L\left(\lambda_{p},\;X_{p}\right) \end{array}\right] \end{array}$$

where *L*(·, ·) is a criterion that needs to be minimized. A simplification of this case is the *spherical* setting. Only a scalar value λ gets optimized and applied to every column.


(2) $$\begin{array}{r} \lambda^{*}=\underset{\lambda }{\mathrm{argmin}}\overset{i=p}{\underset{i=1}{\sum }}L\left(\lambda ,\;X_{i}\right) \end{array}$$

The most general case is called *full* and optimizes.


(3) $$\begin{array}{r} \Lambda^{*}=\left[\begin{array}{c} \lambda_{1}^{*}\\ \lambda_{2}^{*}\\ ...\\ \lambda_{p}^{*} \end{array}\right]=\left[\begin{array}{c} \underset{\lambda_{1}}{\mathrm{argmin}}L\left(\Lambda ,\;X\right)\\ \underset{\lambda_{2}}{\mathrm{argmin}}L\left(\Lambda ,\;X\right)\\ ...\\ \underset{\lambda_{p}}{\mathrm{argmin}}L\left(\Lambda ,\;X\right) \end{array}\right] \end{array}$$

## Motivation

To demonstrate the influence of the Box-Cox transformation, a stratified cross validation with 10 folds and 5 repetitions was executed on various artificial 2-dimensional binary classification tasks with varying Λs. For each direction, *i*∈{1, 2}, λ_*i*_ was distributed evenly in the interval [−5, 5] with a spacing of 1. Hence, 11 × 11 accuracy estimates were conducted. Accuracy measurements were carried out for the different classifiers described in Table 1, as implemented in the Python library scikit-learn (Pedregosa et al., 2011). Additionally, the corresponding acronyms are given. Unless otherwise stated, the default parameters were used, and if provided, random seeds/states were set to 42. Python version 3.6.0, scikit-learn version 0.24.2, NumPy version 1.19.5, and SciPy version 1.5.4 were used.

**Table 1:** Evaluated classifiers for a grid exploration and to test the proposed optimization method on real-world data: Details can be found in Pedregosa et al. (2011).

**Classifier**	**Description**
Linear	Linear classifier with Perceptron loss and trained with stochastic
	gradient descent
KNN	Nearest neighbors voting with number of neighbors *k* = 5
Bayesian	Gaussian naive Bayes classifier
SVC	C-Support Vector Classification with radial basis function kernel
NN	Multi-layer neural network with 2 hidden layers, 10 neurons each,
	relu activation and cross entropy loss

**Figure 1:**
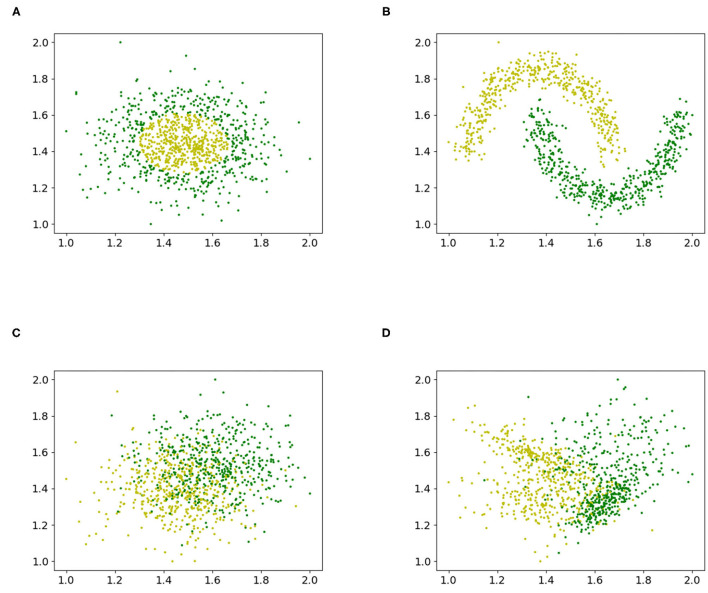
Various artificial binary classification problems were created to study the influence of the Box-Cox transformation with a grid exploration. **(A)** Gaussian quantiles, **(B)** interleaving half circles, **(C)** isotropic Gaussian blobs, and **(D)** random dataset.

Figure 1 shows the different datasets that were used to study the accuracy for different values of Λ.

**Figure 2:**
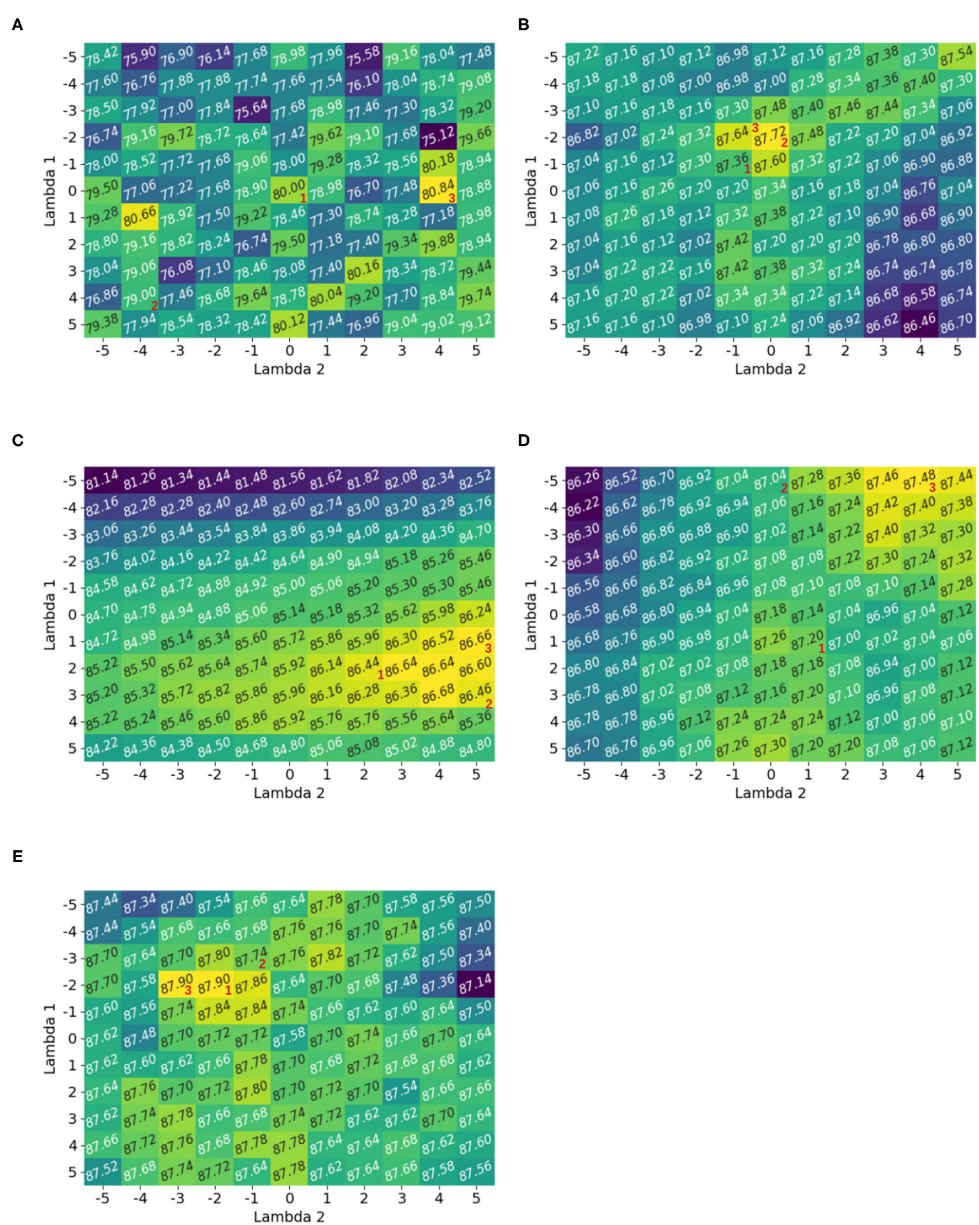
Accuracy heatmaps generated by Algorithm 1 for a random dataset. The numbers 1, 2, and 3 correspond to the optimal solution for the *spherical, diagonal*, and *full* optimization. If there are multiple solutions then only one possibility is shown. It was observed that the optimal parameter choice for the Box-Cox transformation depends on the classifier. The heatmaps showed multiple local maxima and *full* optimization led to the best optimization result. **(A)** Linear classifier, **(B)** KNN classifier, **(C)** Bayesian classifier, **(D)** SVC classifier, and **(E)** NN classifier.

Figure 2 shows the accuracy measurements for the exhaustive grid exploration of Λ on the random classification dataset Figure 1D. The corresponding pseudo-code is given in Algorithm 1. Before applying the Box-Cox transformation, all datasets were preprocessed with a range standardization between 1 and 2. This was done to show the exclusive behavior of the Box-Cox transformation without the influence of other effects; however, the transformation needed positive data. The upper range bound ensured that the features did not explode when transformed with a larger Λ. The results of the Box-Cox transformation were also standard scaled before being given to the classifiers.

**Table:** **Algorithm 1** : 2D Accuracy Gridexploration

*D*: dataset (*X, Y*)
$L_{1}\leftarrow \left\{-5,\;-4,\;\ldots ,\;4,\;5\right\}$
$L_{2}\leftarrow \left\{-5,\;-4,\;\ldots ,\;4,\;5\right\}$
*repetitions*←5
*kfolds*←10 ⊳ number of folds in crossvalidation
*C*: classifier
*A*: matrix to store accuracies
for each item λ_1_ in $L_{1}$ **do**
for each item λ_2_ in $L_{2}$ **do**
*a*←0 ⊳ current accuracy
for *rep* = 1 to*repetitions* **do**
for *I*_*train*_, *I*_*test*_ in cvpartition(*D*, kfold) **do**
$X^{*}=\text{boxcox}\left(X,\left[\lambda_{1},\lambda_{2}\right]\right)$

$X_{train}\leftarrow X^{*}\left[I_{train}\right]$ ⊳ split data
*Y*_*train*_←*Y*[*I*_*train*_]
$X_{test}\leftarrow X^{*}\left[I_{test}\right]$
*Y*_*test*_←*Y*[*I*_*test*_]

Scaler = Standard_Scaler()
*X*_*train*_= Scaler.fit_transform(*X*_*train*_) ⊳ train model
*C*_*t*_ = train(*C, X*_*train*_, *Y*_*train*_)

*X*_*test*_= Scaler.transform (*X*_*test*_) ⊳ evaluate model
*P* = predict(*C*_*t*_, *X*_*test*_)
*a*←*a* + accuracy (*P, Y*_*test*_)
end **for**
end **for**
$A\left[\lambda_{1},\;\lambda_{2}\right]=\frac{a}{repetitions*kfolds}$
end **for**
end **for**

It was observed that the different heatmaps were not similar; hence, the Box-Cox transformation was dependent on the classifier itself. For example, Λ = [−5, 4] gave the best performance for the SVC classifier, but it was almost the worst for the neural network. While Λ = [1, 5] was the best for the Bayesian classifier, it was bad for the KNN classifier. This suggests that the optimization of the Box-Cox transformation was not only dependent on the data but also on the classifier. This observation was also made by Bicego and Baldo (2016).

The heatmaps also showed multiple local maxima. Hence, the optimization should be non-convex. Similar observations were made for the other datasets, and the corresponding heatmaps are provided in Appendix A.

Finally, it was obvious that the *full* optimization gave better results than the *spherical* and *diagonal* settings. The possible *spherical* configurations were seen on the diagonal of the heatmap (e.g., Λ∈{[−5, −5], [−4, −4], …, [5, 5]}). The diagonal can be illustrated by first fixing one direction λ_*i*_ = 1 and optimizing in the other direction and then vice versa. Possible optimal solutions for the *spherical, diagonal*, and *full* optimization were indicated with corresponding numbers 1, 2, and 3. If there were multiple options for the optimal solution in one direction for *diagonal* optimization, then the case that led to higher final optimization accuracy was used.

Table 2 summarizes the accuracy heatmaps for all four datasets in Figure 1. It shows the performance before applying the Box-Cox transformation and after applying the Box-Cox transformation with the best reported configuration of Λ. The numbers are rounded to the first decimal point. The accuracy before applying the Box-Cox transformation corresponds to a Box-Cox transformation with Λ = [1, 1] because this only shifts the data by 1 in each direction and therefore does not influence the classification result.

**Table 2:** Accuracy of five classifiers before and after applying Box-Cox transformation using three optimization strategies.

**Classifier**	**Acc before [%]**	**Acc after [%]**	**Full (δ) [%]**	**Spherical (δ) [%]**	**Diagonal (δ) [%]**
**Gaussian quantiles**					
Linear	49.0	54.1	6.1	5.2	5.2
KNN	96.2	96.6	0.4	0.1	0.3
Bayesian	96.8	96.9	0.1	0.0	0.1
SVC	98.8	99.2	0.5	0.1	0.2
NN	98.9	99.2	0.3	0.1	0.1
**Interleaving half circles**					
Linear	83.5	84.8	1.3	0.4	−0.4
KNN	100.0	100.0	0.0	0.0	0.0
Bayesian	87.4	89.3	1.9	0.0	1.9
SVC	99.7	99.7	0.1	0.0	0.0
NN	99.9	100.0	0.0	0.0	0.0
**Isotropic Gaussian blobs**					
Linear	68.1	70.5	2.4	1.9	−0.8
KNN	74.6	75.2	0.5	0.5	0.2
Bayesian	76.1	76.4	0.3	0.0	−1.0
SVC	75.7	76.1	0.4	0.4	0.1
NN	76.0	76.5	0.5	0.3	0.3
**Random dataset**					
Linear	77.3	80.5	3.5	2.7	1.7
KNN	87.2	87.7	0.5	0.1	0.5
Bayesian	85.9	86.6	0.8	0.6	0.6
SVC	87.2	87.5	0.3	0.0	−0.2
NN	87.7	87.9	0.2	0.2	0.0

*Column Acc after corresponded to full optimization, which was observed as the optimal optimization. Spherical was able to get smaller but also consistent improvements. Diagonal achieved some gains but sometimes decreased the accuracy*.

It was observed that the linear classifier benefited most from the Box-Cox transformation. The other classifiers also benefited, unless the classification result was almost perfect before applying the transformation (KNN and NN in the interleaving half circles dataset). Thus, Box-Cox transformation consistently improved the classification result.

It was also seen that, mostly, *spherical* optimization did not achieve the same improvements as *full* optimization. This is expected because of the lower number of parameters. In contrast, however, *diagonal* optimization resulted in even worse accuracies. This was observed, for example, for the linear classifier in the interleaving half circles dataset. Fixing in one direction and optimizing in the other direction resulted in an increase in accuracy (fixing λ_1_ = 1 led to λ_2_ = 4 with an improvement of 0.68%, and fixing λ_2_ = 1 led to λ_1_ = −3 with an improvement of 0.54%). However, combining the independent results led to Λ = [−3, 4] and a loss of accuracy of −0.36%. This can get arbitrarily bad because the outcome of a combination of the independent optimizations was unknown in advance.

To further study the behavior of the different optimization methods, the random dataset Figure 1D was generated 10 times with different random seeds and the accuracy for each optimization method was measured for the five classifiers given in Table 1 with a stratified cross validation with 10 folds and 5 repetitions. The average of the accuracy is given in Table 3.

**Table 3:** Average accuracy of five classifiers before and after applying Box-Cox transformation using three optimization strategies for 10 times regenerated random dataset Figure 1D with different random seeds.

**Classifier**	**Acc before [%]**	**Acc after [%]**	**Full (δ) [%]**	**Spherical (δ) [%]**	**Diagonal (δ) [%]**
Linear	84.1	86.7	2.6	1.8	0.3
KNN	92.0	92.4	0.4	0.2	0.3
Bayesian	89.0	90.2	1.3	0.8	1.0
SVC	91.9	92.3	0.4	0.2	0.2
NN	92.3	92.6	0.3	0.1	0.1

*Column Acc after corresponded to full optimization, which was observed as the optimal optimization. Spherical was able to get smaller but also consistent improvements. Diagonal achieved smaller gains*.

The *Full* optimization led consistently to the highest improvement in accuracy. Both *Spherical* and *Diagonal* optimization achieved an improvement for all classifiers. *Diagonal* optimization was better or equal than *Spherical* optimization for all classifiers except for the linear classifier.

## Model and Optimization

The previous section showed that *full* optimization led to the best improvements. It was also demonstrated that the optimization was dependent on the classifier. Therefore, we propose a procedure for classifier-dependent multi-dimensional non-convex optimization. First, the general setup is described. Then, naive optimization is introduced. This was used as a baseline but suffered from the curse of dimensionality. Next, an iterative optimization is described that solved the dimensionality problem. Subsequently, various techniques for improving the iterative procedure are presented.

The general setup that was used with different optimization techniques consisted of a training function and a predicting function. It is shown in Algorithm 2. First, a model was trained to find the optimal parameter, Λ, for the Box-Cox transformation with a given classifier. Then the predicting function was used with the optimized Box-Cox parameter, Λ, to create predictions.

**Table:** **Algorithm 2** : Setup

*X*_*train*_: training features
*Y*_*train*_: training class labels
*X*_*test*_: testing features
*C*: classifier/model to optimize for
*M*: min-max scaler
*S*: standard scaler

Λ, *C, M, S*← fit_model(*X*_*train*_, *Y*_*train*_, *C*)
*Y*_*test*_← prediction(*X*_*test*_, Λ, *C, M, S*)

The training procedure is given in Algorithm 3. It requires the features, the corresponding class labels, a classifier, and an optimization procedure for Λ. Suitable optimization procedures are given in Algorithm 5 (restricted to 2-dimensional data) and Algorithm 6 with further improvements for the latter in 6.1, 6.2, and 6.3. It first scaled the data into the range [1, 2] to ensure that the features were positive so that the Box-Cox transformation could be applied, and to ensure that the features did not explode at a larger Λ. Then, an optimization procedure was applied to find suitable values for Λ. As described in the previous section, this was dependent on the classifier itself. Next, the Box-Cox transformation was applied to the features with the optimized Λ. Then, the data were standard scaled to help classifiers that depended on a distance measure. Finally, the classifier was trained.

**Table:** **Algorithm 3** : fit_model(*X, Y, C*)

1: *X*: features
2: *Y*: corresponding class labels
3: *C*: classifier to optimize for
4: *Opt*: optimization procedure for optimizing Λ
5: *M*: min-max scaler into the range [1, 2]
6: *S*: standard scaler
7:
8: *X*_*M*_←*M*.*fit*_*transform*(*X*) ⊳ fit min-max scaler and apply it
9: Λ←*Opt*(*X*_*M*_, *Y, C*) ⊳ find optimized Λ
10: ⊳ (e.g. Algorithm 6, 6.1, 6.2, 6.3, and 5 for 2D)
11: *X*_*B*_← boxcox(*X*_*M*_, Λ) ⊳ apply Box-Cox transformation
12: *X*_*S*_←*S*.*fit*_*transform*(*X*_*B*_) ⊳ fit standard scaler and apply it
13: *C*.train(*X*_*S*_, *Y*) ⊳ train classifier
14:
15: return Λ, *C, M, S*

The prediction procedure is presented in Algorithm 4. It required the features, Λ, which was optimized during training, a fitted classifier, a fitted min-max scaler, and a fitted standard scaler. First, the method min-max scaled the data, then applied the Box-Cox transformation with the given Λ, then used standard scaling, and finally predicted the labels with the given classifier.

**Table:** **Algorithm 4** : prediction(*X*, Λ, *C, M, S*)

*X*: features
Λ: optimized parameters of Box-Cox transformation
*C*: trained classifier
*M*: fitted min-max scaler
*S*: fitted standard scaler

*X*_*M*_←*M*.*transform*(*X*) ⊳ apply fitted min-max scaler
*X*_*B*_← boxcox(*X*_*M*_, Λ) ⊳ apply Box-Cox transformation
*X*_*S*_←*S*.*transform*(*X*_*B*_) ⊳ apply fitted standard scaler
*Y*←*C*.predict(*X*_*S*_) ⊳ predict labels with trained classifier

return *Y*

To follow the previously introduced notation in this paper, the optimization criteria *L*(·, ·) is defined as 1−*ACC*, which maximizes accuracy *ACC* by minimizing the 1−*ACC* optimizer. The first optimization procedure that was used in the training function was a grid search. This means that a set of possible values for every λ_*i*_ was specified. Then, the optimization tried all combinations. This was an exhaustive search and assuming model fitting and predicting as constant, it runs in polynomial time *O*(*L*^*p*^) where *L* is the number of possible values and *p* is the number of features. Therefore, the grid search suffered from the curse of dimensionality. For example, trying 10 values for 10 features requires 10 billion evaluations. Therefore, this became quite infeasible. Nevertheless, it was used as a reference model for lower dimensional datasets. The pseudo-code for this method for the 2-dimensional case was given in Algorithm 5 and was directly used as optimization for training in Algorithm 3 in line 9.

**Table:** **Algorithm 5** : 2D grid search(*X, Y, C*)

*X*: features
*Y*: corresponding class labels
*C*: classifier to optimize for
*S*: standard scaler
Λ_*opt*_: optimized Box-Cox parameters
*grid*←{−5, −4, …, 4, 5} ⊳ candidate values for each direction
*A*←0 ⊳ best accuracy obtained during search

for each λ_1_ in *grid* **do**
for each λ_2_ in *grid* **do**
Λ_*tmp*_←[λ_1_, λ_2_]
*X*_*B*_← boxcox(*X*, Λ_*tmp*_) ⊳ apply Box-Cox transformation
*X*_*S*_←*S*.*fit*_*transform*(*X*_*B*_) ⊳ fit standard scaler and apply it
*C*.train(*X*_*S*_, *Y*) ⊳ train classifier
*P*←*C*.predict(*X*_*S*_) ⊳ evaluate classifier
*A*_*tmp*_← accuracy(*P, Y*) ⊳ evaluate accuracy

if *A*_*tmp*_>*A* **then** ⊳ update Λ_*opt*_ if accuracy is improved
*A*←*A*_*tmp*_
Λ_*opt*_←Λ_*tmp*_
end **if**

end **for**
end **for**
return Λ_*opt*_

To solve the dimensionality problem of a grid search, we proposed an iterative optimization. First, an initial point, *G*∈ℝ^*p*^, for Λ was specified. Then, starting from this point, all directions were fixed except for one. The not-fixed direction was optimized with a 1−dimensional grid search. Therefore, a set of candidate values for the search needed to be defined. Comparing the possible values and selecting the one that gave the highest improvement led to optimization in the first direction. Then, the next direction was unfixed and all other directions were fixed. Again, the best value was selected with a 1-dimensional grid search. This procedure was repeated until all directions were optimized once. This was referred to as one *epoch*. After that, the same procedure restarted with the previously optimized solution instead of the initial point *G*. The pseudocode for this iterative optimization was given in Algorithm 6 and will be referred to as *Iterative grid search*. It was directly used as an optimization procedure for training in Algorithm 3 in line 9. Assuming model fitting and predicting as constant, the advantage of this method is that it scaled linearly *O*(*epochs*·*p*·*gridsize*) in the number of features *p*, where gridsize denotes the number of points used for the 1-dimensional grid search. This procedure had three hyperparameters that influenced the result (initial starting point *G*, number of epochs, and the grid).

**Table:** **Algorithm 6** : Iterative grid search(*X, Y, C*)

⊳ full optimization to get optimal Λ vector
1: *X*: features
2: *Y*: corresponding class labels
3: *C*: classifier to optimize for
4: *p*: number of features/directions
5: *S*: standard scaler
6: *G*: initial starting point
7: Λ_*opt*_←*G* ⊳ optimized Box-Cox parameter
8: *grid*←{−5, −4, …, 4, 5} ⊳ candidate values for each direction
9: *epochs*←*e* ⊳ number of epochs
10: *A*←0 ⊳ best accuracy obtained during search
11:
12: for *epoch* = 1 to *epochs* **do**
13: for *dir* = 1 to *p* **do**
14: Λ_*tmp*_←Λ_*opt*_
15: for each λ_*i*_ in *grid* **do**
16: Λ_*tmp*_[*dir*]←λ_*i*_ ⊳ change one direction
17: *X*_*B*_← boxcox(*X*, Λ_*tmp*_) ⊳ apply Box-Cox transformation
18: *X*_*S*_←*S*.*fit*_*transform*(*X*_*B*_) ⊳ fit standard scaler and apply it
19: *C*.train(*X*_*S*_, *Y*) ⊳ train classifier
20: *P*←*C*.predict(*X*_*S*_) ⊳ evaluate classifier
21: *A*_*tmp*_← accuracy(*P, Y*) ⊳ evaluate accuracy
22:
23: if *A*_*tmp*_>*A* **then** ⊳ update Λ_*opt*_ if accuracy is improved
24: *A*←*A*_*tmp*_
25: Λ_*opt*_←Λ_*tmp*_
26: end **if**
27:
28: end **for**
29: end **for**
30: end **for**
31: return Λ_*opt*_

**Figure 3:**
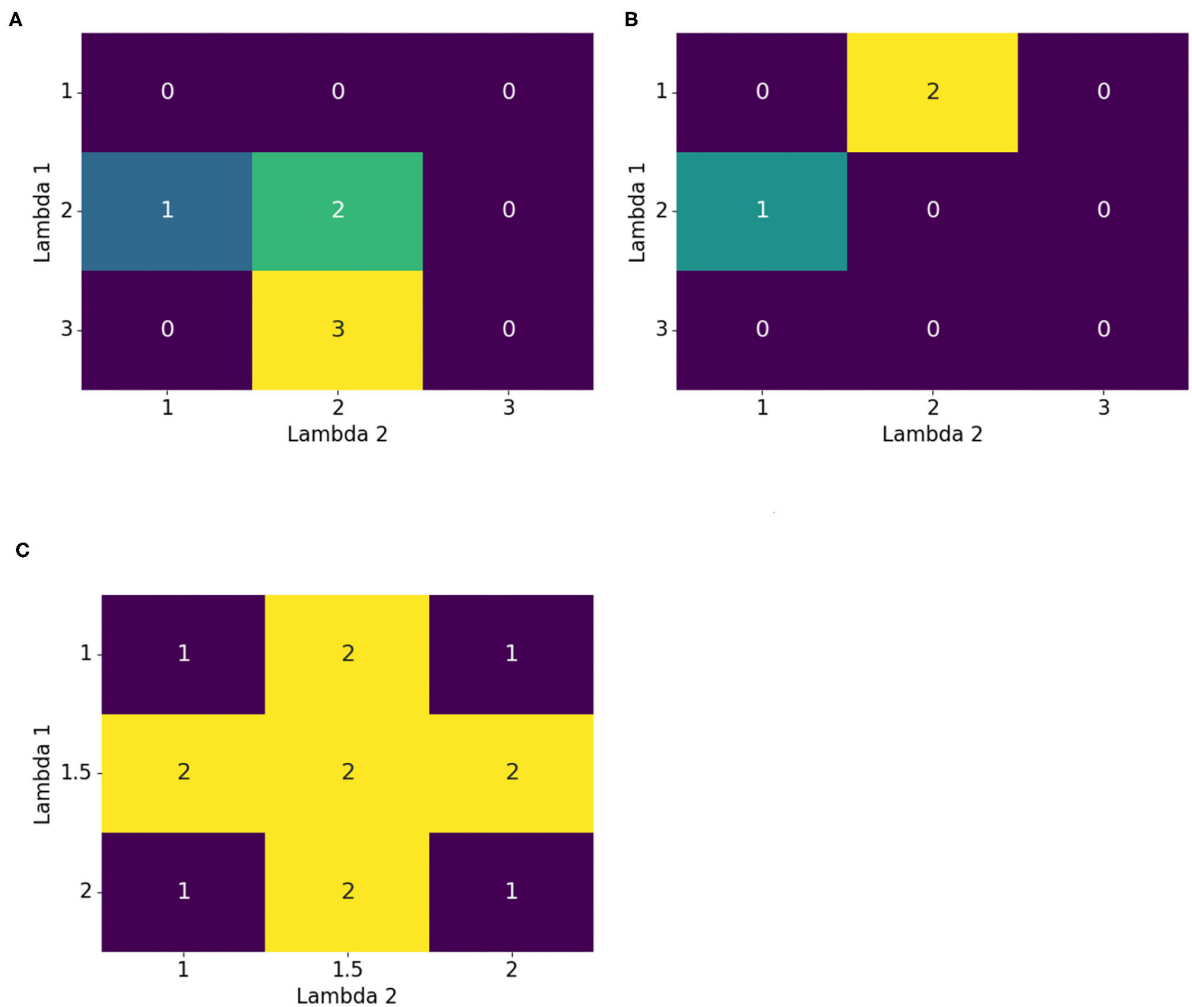
Motivations for improvements to the iterative method. Multiple epochs helped to further advance the optimization to the maximum. Multiple starting points and shuffling were introduced for escaping or avoiding a local maximum, and a finer grid provided the ability to explore hidden details. **(A)** Multiple epochs, **(B)** Multiple starting points and shuffling optimization order, and **(C)** Finer grid.

Figure 3A shows why multiple epochs were beneficial. From the initial point *G* = [1, 1], first optimizing vertically in the λ_1_ direction, and then horizontally in the λ_2_ direction, gave an optimal value of 2 for Λ = [2, 2]. If another epoch, and thus another optimization in both directions, was added, the global optimal solution 3 at Λ = [3, 2] was obtained. Therefore, multiple epochs helped to find better optimization results.

Another useful improvement was to restart the optimization with another initial point. Figure 3B illustrates this. Starting at *G* = [1, 1] and first optimizing vertically and then horizontally resulted in Λ_*opt*_ = [2, 1]. This cannot be further optimized with the given iterative method. Unfortunately, there was a better solution at Λ = [1, 2]. If, for example, the optimization started at *G* = [2, 2], the global optimal solution could be attained. Therefore, it was beneficial to restart the optimization procedure with multiple initial points. Corresponding modifications to the *Iterative grid search* optimization are found in Algorithm 6.1. It introduced the *shift_epoch* as a new hyperparameter that determined after how many *epochs* a new starting point *G* was generated.

**Table:** **Algorithm 6.1**: Shift(*X, Y, C*)

⊳ iterative grid search with multiple start points

… ⊳ same as Algorithm 6 line 1−10
*shift*_*epoch*←*s* ⊳ number of epochs until new starting point
*shift*← False ⊳ Boolean flag to indicate a new starting point

for *epoch* = 1 to *epochs* **do**

if *epoch* mod *shift*_*epoch* = =0 **then**
*G*← generate_initial_point()
*shift*← True
end **if**

if *shift* **then**
Λ_*tmp*_←*G*
else
Λ_*tmp*_←Λ_*opt*_
end **if**

for *dir* = 1 to *p* **do**
for each λ_*i*_ in *grid* **do**
… ⊳ same as Algorithm 6 line 16−21
if *A*_*tmp*_>*A* **then** ⊳ update Λ_*opt*_ if accuracy is improved
*A*←*A*_*tmp*_
Λ_*opt*_←Λ_*tmp*_
*shift*← False
end **if**

end **for**
end **for**
end **for**
return Λ_*opt*_

The previous problem could also be solved by changing the order of the optimization directions. So far, the directions have been optimized numerically; that is, first, λ_1_ was optimized, then λ_2_ and so on. Starting (in Figure 3B) again at the initial point *G* = [1, 1], instead of first optimizing in the vertical direction, optimization was done first in the horizontal direction. This directly found the global solution. Hence, shuffling the order of directions for optimization was also helpful. The corresponding changes to *Iterative grid search* are found in Algorithm 6.2. Again, there was a new hyperparameter *shuffle_epoch* that determined after how many *epochs* the optimization order got shuffled.

**Table:** **Algorithm 6.2** : Shuffle(*X, Y, C*)

⊳ iterative grid search with changing order of the optimization directions

… ⊳ same as Algorithm 6 line 1−10
*dir*_*order*←[1, 2, …, *p*] ⊳ order of directions for optimization
*shuffle*_*epoch*←*h* ⊳ number of epochs until the order
⊳ of direction gets shuffled

for *epoch* = 1 to *epochs* **do**

if *epoch* mod *shuffle*_*epoch* = =0 **then**
shuffle(*dir*_*order*)
end **if**

for each *dir* in *dir*_*order* **do**
… ⊳ same as Algorithm 6 line 14−28
end **for**
end **for**
return Λ_*opt*_

Lastly, it might be possible to find a better solution if the grid search is denser. Figure 3C demonstrates this. If the grid only used integer values, then it was impossible to find one of the global optimal solutions = 2. Hence, the grid should be refined to 0.5 increments. Unfortunately, this doubled the computational demand. Another refinement may further improve the result but increase the computational demand even more. One solution to circumvent the increasing computational costs, was to use local refinement. This means that the grid became locally denser and smaller. *Iterative grid search* uses the same global grid for every 1-dimensional grid search (e.g. {−5, −4, …, 4, 5}). To get a finer grid, but with the same number of points, the grid needed to be attached locally to the current Λ_*tmp*_. Since the number of grid points ought to remain the same and the grid became denser, it spanned a smaller range of values. For example, starting with the grid {−5, −4, …, 4, 5} and then doubling the resolution led to the following grid {−2.5, −2, …, 2, 2.5}. Both have the same number of points. Instead of testing globally, if any, λ_*i*_∈{−5, −4, …, 4, 5} improved the result, the current optimal solution in this direction was used, and then the refined grid was attached to it. Therefore, it is tested, if any, λ_*i*_∈{λ_*tmp, i*_−2.5, λ_*tmp, i*_−2, …, λ_*tmp, i*_+2, λ_*tmp, i*_+2.5} improved the accuracy. To take advantage of both global and local optimization, a global search was used at the beginning of the optimization to capture the full search domain. After some epochs, a local refinement was used to obtain a finer search space. With this modification, the computational cost remained the same. Additionally, it allowed for more and finer candidate values that could result in improvement. Incorporating this method into *Iterative grid search* is shown in Algorithm 6.3. As before, an additional hyperparameter *finer_epoch* was introduced to specify after how many *epochs* the grid was refined.

**Table:** **Algorithm 6.3** : Finer(*X, Y, C*)

⊳ iterative grid search with a refined grid

… ⊳ same as Algorithm 6 line 1−10
*finer*←0.5 ⊳ refinement of grid
*finer*_*epoch*←*f* ⊳ number of epochs until the grid gets finer
global = 1 ⊳ use global grid search at the beginning

for *epoch* = 1 to *epochs* **do**

if *epoch* mod *finer*_*epoch* = =0 **then**
*global* = 0
*grid*←finer*grid ⊳ element-wise scale each element in grid
end **if**

for *dir* = 1 to *p* **do**
Λ_*tmp*_←Λ_*opt*_
*candidates*←*grid*+(1−*global*)*Λ_*opt*_[*dir*]
for each λ_*i*_ in *candidates* **do**
… ⊳ same as Algorithm 6 line 16−26
end **for**
end **for**
end **for**
return Λ_*opt*_

Additionally, *spherical* and *diagonal* optimizations are given in Algorithms 7, 8. This was used for comparison with the proposed *full* optimization. These two methods were developed on classification accuracy like *full* optimization, rather than statistical evaluation (such as MLE or Fisher criterion) Bicego and Baldo (2016). The reason behind this approach is that the previous study showed that the Box-Cox parameter is classifier dependent.

**Table:** **Algorithm 7** : Spherical grid search(*X, Y, C*)

⊳ full optimization to get optimal Λ vector
1: *X*: features
2: *Y*: corresponding class labels
3: *C*: classifier to optimize for
4: *p*: number of features/directions
5: *S*: standard scaler
6: Λ_*opt*_←0 ⊳ optimized Box-Cox parameter
7: *grid*←{−5, −4, …, 4, 5} ⊳ candidate values
8: *A*←0 ⊳ best accuracy obtained during search
9:
10: for each λ_*i*_ in *grid* **do**
11: Λ_*tmp*_←[λ_*i*_, λ_*i*_, …, λ_*i*_]
12: *X*_*B*_← boxcox(*X*, Λ_*tmp*_) ⊳ apply Box-Cox transformation
13: *X*_*S*_←*S*.*fit*_*transform*(*X*_*B*_) ⊳ fit standard scaler and apply it
14: *C*.train(*X*_*S*_, *Y*) ⊳ train classifier
15: *P*←*C*.predict(*X*_*S*_) ⊳ evaluate classifier
16: *A*_*tmp*_← accuracy(*P, Y*) ⊳ evaluate accuracy
17:
18: if *A*_*tmp*_>*A* **then** ⊳ update Λ_*opt*_ if accuracy is improved
19: *A*←*A*_*tmp*_
20: Λ_*opt*_←Λ_*tmp*_
21: end **if**
22:
23: end **for**
24: return Λ_*opt*_

**Table:** **Algorithm 8** : Diagonal grid search(*X, Y, C*)

⊳ full optimization to get optimal Λ vector
1: *X*: features
2: *Y*: corresponding class labels
3: *C*: classifier to optimize for
4: *p*: number of features/directions
5: *S*: standard scaler
6: Λ_*opt*_←[1, 1, …, 1] ⊳ optimized Box-Cox parameter
7: *grid*←{−5, −4, …, 4, 5} ⊳ candidate values for each direction
8: *A*←0 ⊳ best accuracy obtained during search
9:
10: for *dir* = 1 to *p* **do**
11: Λ_*tmp*_←[1, 1, …, 1]
12: for each λ_*i*_ in *grid* **do**
13: Λ_*tmp*_[*dir*]←λ_*i*_ ⊳ change one direction
14: *X*_*B*_← boxcox(*X*, Λ_*tmp*_) ⊳ apply Box-Cox transformation
15: *X*_*S*_←*S*.*fit*_*transform*(*X*_*B*_) ⊳ fit standard scaler and apply it
16: *C*.train(*X*_*S*_, *Y*) ⊳ train classifier
17: *P*←*C*.predict(*X*_*S*_) ⊳ evaluate classifier
18: *A*_*tmp*_← accuracy(*P, Y*) ⊳ evaluate accuracy
19:
20: if *A*_*tmp*_>*A* **then** ⊳ update Λ_*opt*_ if accuracy is improved
21: *A*←*A*_*tmp*_
22: Λ_*opt*_←Λ_*tmp*_
23: end **if**
24: end **for**
25: end **for**
26: return Λ_*opt*_

## Results

Following the proposed optimization procedure was applied to different real-world datasets. The setup in Algorithm 2 was used which means that Algorithm 3 was used to train the model on the training data with the iterative optimization from Algorithm 6 and the corresponding improvements 6.1, 6.2, and 6.3. Then the performance was measured using the prediction function in Algorithm 4. The examined classifiers are given in Table 1. All results were measured with 10-fold stratified crossvalidation and 5 repetitions. To test the proposed method various settings for the hyperparameters were used. The setup is given in Table 4. Optimization in one direction was done evenly spaced over the interval [−5, 5] and gridsize corresponded to the number of grid points (e.g. gridsize of 11 gave the set {−5, −4, …, 4, 5} as candidate values). The *Iterative grid search* was just iterative optimization without any further improvements described in Algorithm 6. *Shift, Shuffle*, and *Finer* exclusively showed the influence of restarting the optimization with a new starting point given in Algorithm 6.1, changing the order of directions given in Algorithm 6.2, or refining the optimization grid given in Algorithm 6.3. *Combined 1* and *Combined 2* demonstrated how these improvements to the *Iterative grid search* optimization behaved in combination. The initial starting point, *G*∈ℝ^*p*^, for the iterative optimization was chosen in each direction as the MLE. For comparison *spherical* and *diagonal* optimizations are given in Algorithms 7, 8 was also evaluated. Further traditional Box-Cox optimization of the log-likelihood function as in Box and Cox (1964) was applied column-wise. This approach maximized the Gaussianity of each column and is called *MLE* in the following tables.

**Table 4:** Hyperparameter settings to test the iterative optimization on real-world data.

**Name**	**Gridsize**	**Epochs**	**Shift_epoch**	**Shuffle_epoch**	**Finer_epoch**
Iterative grid search	11	4	4	4	4
Shift	11	8	4	8	8
Shuffle	11	8	8	2	8
Finer	11	8	8	8	4
Combined 1	11	16	8	2	4
Combined 2	21	16	8	2	4

###  Sonar Dataset

The sonar dataset had 207 samples and 60 features. The labels were binary and indicated whether the sonar signal was reflected by a rock or metal. The measurements for the accuracy of the repeated cross-validation are given in Table 5. Additional measurements for the F1-score are given in the Appendix in Table B1.

**Table 5:** Improvement δ in accuracy for different iterative optimization settings in the sonar dataset.

	**Linear [%]**	**KNN [%]**	**Bayesian [%]**	**SVC [%]**	**NN [%]**
Base accuracy	75.195	81.343	67.700	84.052	84.024
Diagonal	0.076	0.290	1.829	–0.395	–0.957
Spherical	0.586	0.095	6.067	–0.300	–1.910
MLE	–0.167	0.000	6.443	1.810	–0.291
Iterative grid search (δ)	1.162	1.824	7.919	2.286	–0.386
Shift (δ)	0.976	1.919	7.919	2.381	–0.386
Shuffle (δ)	1.162	1.824	7.919	2.286	–0.386
Finer (δ)	0.876	1.824	7.919	2.286	–0.386
Combined 1 (δ)	0.600	1.838	7.157	2.190	–0.386
Combined 2 (δ)	2.795	3.267	8.100	2.010	–0.486

*The proposed optimization achieved a consistent improvement except for the neural network. The different hyperparameter settings had a varying influence on the classifiers defined in Table 4. Combined 2 improved the linear, KNN, and Bayesian classifier, whereas Shift already delivered the best performance for SVC. Additionally, the proposed optimization achieved higher improvements than Diagonal, Spherical and MLE except for the neural network*.

There was an improvement in accuracy for the proposed optimization for all classifiers except for the neural network. In particular, the Bayesian classifier improved on average by 7.8%. In contrast, the neural network decreased by −0.4% on average. The influence of the changes to the basic iterative optimization in Algorithm 6 with *Shift, Shuffle*, and *Finer* was observed for the linear and KNN classifiers. *Shift* restarted the optimization with a new initial point, and it seemed to decrease the accuracy for the linear classifier but slightly increased it for KNN. In contrast, shuffling the order of the directions during optimization did not result in an advantage for the classifier. Refining the grid after some epochs did not provide an increase in accuracy compared to basic iterative optimization. Combining these methods into one optimization sometimes decreased the performance (SVC) and sometimes increased the performance (KNN). Interestingly, the influence of the *Combined 1* was better for the SVC and the neural network when compared to the *Combined 2*, which had a finer grid for Λ. The opposite was observed for the other classifiers. The *Diagonal, Spherical*, and *MLE* optimization performed worse than the proposed optimization except the *MLE* optimization lead to a smaller decrease in accuracy for the neural network. This behavior was also observed for the F1-score measurements given in Appendix in Table B1.

**Figure 4:**
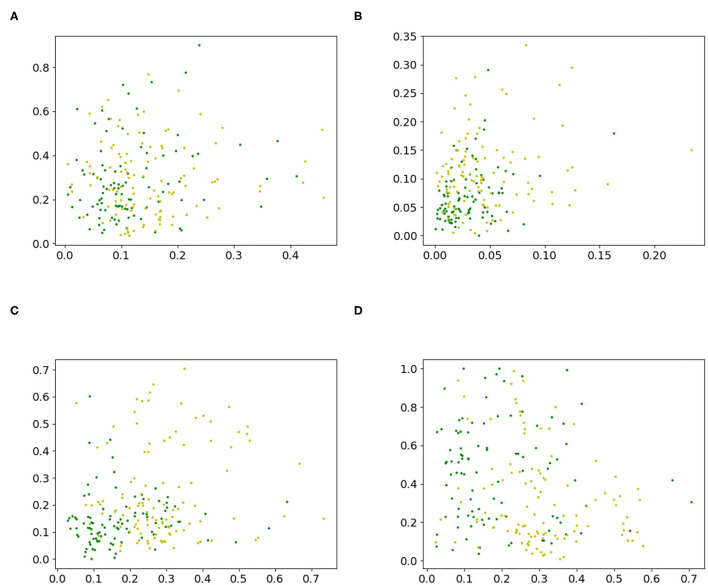
2-D datasets extracted from the sonar dataset to compare proposed iterative optimization with a grid search. **(A)** features 8 and 41, **(B)** features 2 and 48, **(C)** features 11 and 45, and **(D)** features 12 and 36.

A 2-dimensional feature study was also performed. Two 2-dimensional datasets, Figures 4A,B, were created by extracting two random features from the sonar dataset. With a chi-square test, the ranks of the features were calculated. Then, a dataset with the two highest ranking features, Figure 4C and a dataset Figure 4D, with the third and fourth highest ranking features, were built. The performance of a grid search was measured and served as the baseline. This was done by using the training function in Algorithm 3 with 2D grid search from Algorithm 5 as optimization procedure and Algorithm 4 to create predictions. The grid search used the grid {−5, −4, …, 4, 5} in every direction. The datasets are shown in Figure 4, and the results for the accuracy are given in Table 6 and for the F1-scores in Appendix in Table B2.

**Table 6:** Improvement δ in accuracy for different iterative optimization settings on 2-dimensional subsets of the sonar dataset.

	**Linear [%]**	**KNN [%]**	**Bayesian [%]**	**SVC [%]**	**NN [%]**
Features 8 and 41					
Base accuracy	53.576	54.090	57.390	62.224	63.267
Diagonal	–2.952	0.729	2.276	–0.652	–0.100
Spherical	6.148	0.824	4.067	–0.471	0.005
MLE	–0.310	0.643	4.267	0.857	0.014
2D grid search (δ)	7.081	–0.924	3.476	–1.138	–1.243
Iterative grid search (δ)	7.595	–1.195	3.481	–0.195	0.100
Shift (δ)	8.648	–1.005	3.681	–0.767	–0.567
Shuffle (δ)	7.595	–1.195	3.481	–0.195	0.100
Finer (δ)	8.657	–1.200	3.386	–0.386	–0.376
Combined 1 (δ)	8.843	–2.252	3.671	–0.276	–0.376
Combined 2 (δ)	6.743	–1.190	4.357	0.200	–0.671
Features 2 and 48					
Base accuracy	55.948	64.862	62.590	66.538	66.719
Diagonal	3.686	0.267	4.943	0.886	–1.329
Spherical	11.276	–0.424	4.957	1.467	0.214
MLE	–1.186	1.424	5.519	0.019	–0.852
2D grid search (δ)	11.562	0.090	5.224	1.271	–0.086
Iterative grid search (δ)	12.638	–0.014	4.633	0.305	0.105
Shift (δ)	12.343	0.186	4.633	0.305	0.300
Shuffle (δ)	12.638	–0.014	4.633	0.305	0.105
Finer (δ)	12.724	0.567	4.448	0.800	0.105
Combined 1 (δ)	12.052	0.476	4.543	0.510	0.200
Combined 2 (δ)	11.571	–0.100	4.543	0.705	-0.557
Features 11 and 45					
Base accuracy	63.995	71.371	64.805	73.486	72.995
Diagonal	1.943	–0.176	8.310	–1.148	0.319
Spherical	8.433	–0.186	7.362	–0.186	–0.757
MLE	0.205	–2.124	5.910	–0.281	0.400
2D grid search (δ)	9.495	–0.186	8.886	–0.095	–1.162
Iterative grid search (δ)	8.838	–0.957	9.462	0.090	–1.257
Shift (δ)	9.029	–0.662	9.367	–0.004	–1.062
Shuffle (δ)	8.838	–0.957	9.462	0.090	–1.257
Finer (δ)	9.124	–0.567	9.562	0.190	–1.152
Combined 1 (δ)	8.838	–0.567	9.562	0.190	–1.252
Combined 2 (δ)	9.319	–0.752	9.948	0.190	–1.048
Features 12 and 36					
Base accuracy	59.510	70.443	70.705	68.876	69.824
Diagonal	3.662	–1.271	-0.595	1.448	0.967
Spherical	10.286	–1.381	1.138	0.776	0.200
MLE	5.343	–1.281	–0.290	1.157	1.452
2D grid search (δ)	12.267	0.681	–0.695	1.833	0.386
Iterative grid search (δ)	12.133	1.733	–1.543	0.767	1.062
Shift (δ)	11.957	1.733	–1.543	0.867	0.776
Shuffle (δ)	12.133	1.733	–1.543	0.767	1.062
Finer (δ)	12.443	1.924	–1.929	0.771	0.686
Combined 1 (δ)	12.157	2.019	–2.024	1.157	1.462
Combined 2 (δ)	12.652	2.010	–1.914	0.676	1.552

*In 16 out of the 20 feature classification cases (4 tests × 5 classifiers), an improvement was achieved compared to the base accuracy. In 13 out of the 20 cases, iterative optimization was better than a 2D grid search. Additionally, it was better than Diagonal optimization in 13 out of 20 cases and better than Spherical and MLE optimization in 14 out of 20 cases for each. The influence of the hyperparameter settings is data and classifier dependent*.

The iterative method delivered an improvement of the base accuracy in 16 out of the 20 cases for at least one hyperparameter setting. Only the accuracy for the KNN classifier for features [8, 41] and [11, 45], the Bayesian classifier for features [12, 36], and the neural network for features [11, 45] could not be improved. Using the hyperparameter setting that resulted in the lowest loss for each case, the highest decrease in accuracy was only −1.543% (Bayesian classifier for features [12, 36]). In contrast, the best hyperparameter setting achieved a gain of 12.724% (linear classifier for features [12, 36]). Comparing the proposed iterative method to a grid search, the iterative optimization achieved better results for all classifiers for features [8, 41] for at least one hyperparameter setting except for the KNN classifier. Further, it provided gains for the linear, KNN, and neural network for features [2, 48], Bayesian, SVC, and neural network for features [11, 47], and linear, KNN and neural network for features [12, 36]. Hence, in 13 out of the 20 cases, iterative optimization resulted in better performance than *2D grid search*. The gain in accuracy for the linear classifier was always positive. This also holds for the Bayesian classifier, except for features [12, 36]. The KNN classifier fluctuated around zero. Sometimes the iterative method cannot achieve any improvement for all tested hyperparameters setting (features [8, 41] and [11, 45]), and sometimes it was able to improve the result. For the SVC, there was always at least one hyperparameter choice that led to an improvement. The same can be observed for the neural network, except for features [11, 45]. Nevertheless, it resulted in a smaller loss of accuracy than grid search for the *Shift, Finer*, and *Combined 2* cases. For all classifiers and datasets, there was often a set of hyperparameters that improved the result of the *Iterative grid search* optimization. Additionally, the influence of *Shift* and *Finer* compared to *Iterative grid search* was sometimes positive and sometimes negative. This varied between classifiers applied to the same dataset, for example, for features [8, 41] *Finer* increased the accuracy for the linear classifier but decreased the accuracy for the KNN classifier. Further, it varied between datasets for the same classifier, for example, the performance of the linear classifier for *Shift* increased for features [8, 41] but decreased for features [2, 48]. The same observation can be made for *Combined 1* and *Combined 2*. *Shuffle* did not influence accuracy compared to *Iterative grid search*. The proposed method achieved better results than *Diagonal* optimization in 13 out of 20 cases. For *Spherical* and *MLE* optimization the results were better in 14 out of 20 cases for both. The measurements for the F1-score are given in Table 6. The proposed optimization achieved a better F1-score compared to the base accuracy in 12 out of 20 cases, compared to *2D grid search* in 14 out of 20 cases, compared to *Diagonal* optimization in 13 out of 20 cases, compared to *Spherical* in 16 out of 20 cases and compared to *MLE* optimization in 12 out of 20 cases.

###  Breast Cancer Dataset

This dataset consisted of 569 samples and 30 features. It was a binary classification that distinguished between benign and malignant fine needle aspirate (FNA) samples. The influence of the iterative method on accuracy is given in Table 7. Additionally, measurements for the F1-score are given in the Appendix in Table C3.

**Table 7:** Improvement δ in accuracy for different iterative optimization settings on the breast cancer dataset.

	**Linear [%]**	**KNN [%]**	**Bayesian [%]**	**SVC [*%*]**	**NN [%]**
Base accuracy	96.487	96.838	93.289	97.539	98.103
Diagonal	0.317	–0.140	1.232	0.352	–0.246
Spherical	0.422	0.000	1.443	0.176	–1.230
MLE	–0.177	0.279	1.477	0.246	–0.281
Iterative grid search (δ)	0.175	0.245	1.371	0.211	–0.598
Shift (δ)	0.175	0.069	1.229	0.105	–0.773
Shuffle (δ)	0.175	0.245	1.336	0.211	–0.598
Finer (δ)	0.316	0.245	1.371	0.211	–0.598
Combined 1 (δ)	0.069	0.315	1.336	0.211	–0.563
Combined 2 (δ)	0.239	0.140	1.442	0.211	–0.669

*Every classifier benefited from the proposed optimization apart from the NN. Finer improved the linear classifier the most, Combine 1 the KNN, Combined 2 the Bayesian. Therefore, the best hyperparameter setting was dependent on the classifier. Diagonal optimization was better than the proposed optimization for the linear classifier, SVC and NN. Spherical optimization was better for the linear and Bayesian classifier. MLE optimization was better for the Bayesian classifier, SVC and NN*.

A similar observation to the sonar dataset was evident. There was an improvement for all classifiers except for the neural network. Again, the Bayesian classifier improved the most, and the influence of the different hyperparameter settings on the SVC was almost static. In comparison to *Iterative grid search, Shift* decreased the performance for all classifiers. Extending the *Iterative grid search* optimization with *Shuffle* had a small negative effect on the Bayesian classifier but did not change the accuracies of the other classifiers. *Finer* improved the results for the linear classifier but did not affect it for the other classifiers. *Combined 1* improved the results of the KNN and neural network classifier compared to the *Iterative grid search* optimization. In contrast, *Combined 2* only improved the neural network. The proposed optimization achieved an improvement of accuracy for the KNN and Bayesian classifier compared to *Diagonal* optimization, for the KNN, SVC, and NN compared to *Spherical* optimization, and for the linear classifier and KNN compared to the *MLE* optimization. The same was observed for the F1-scores in Appendix in Table C3 except that the proposed optimization achieved additionally an improvement for the linear classifier compared to the *Diagonal* optimization.

**Figure 5:**
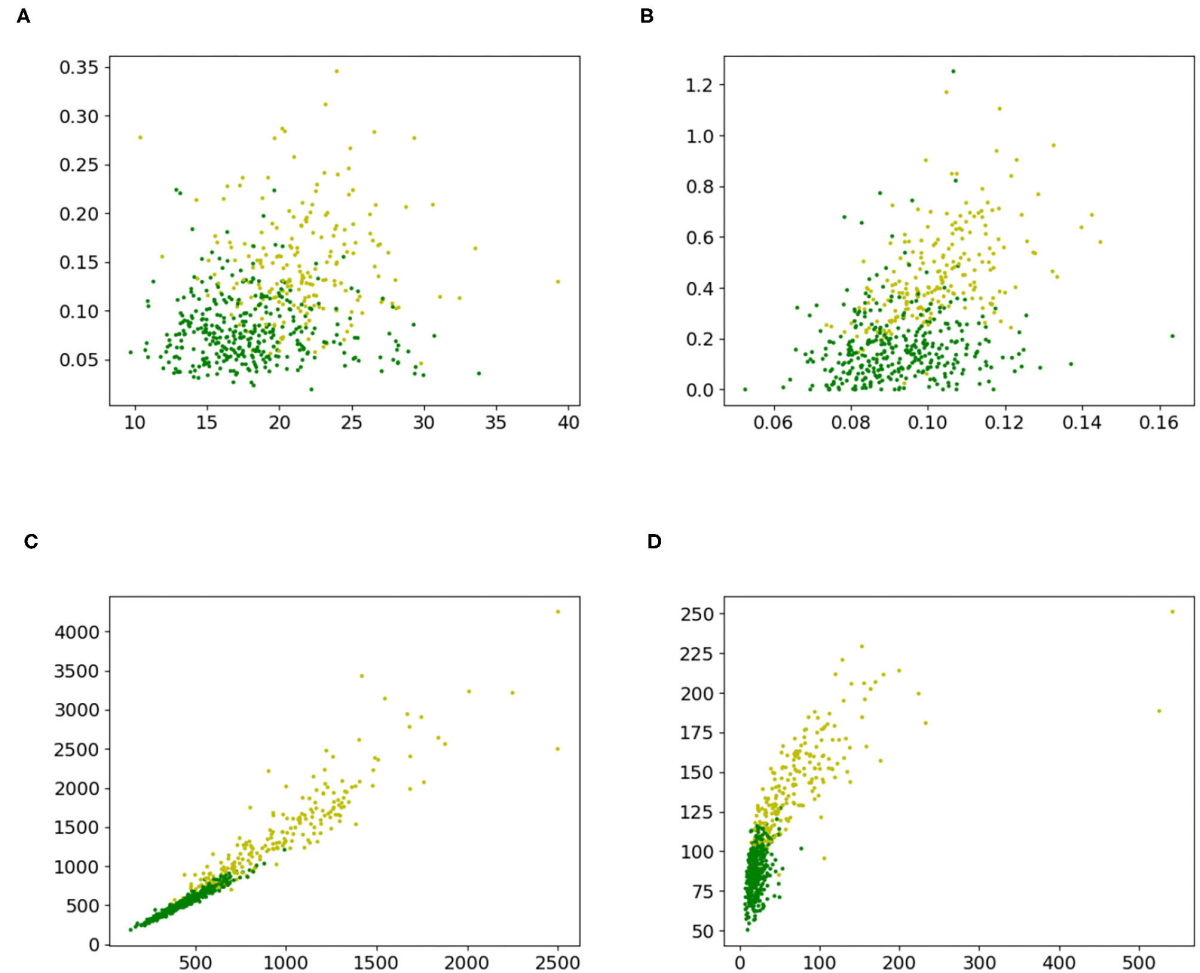
2-D datasets extracted from the breast cancer dataset to compare proposed iterative optimization with a grid search. **(A)** Features 2 and 6, **(B)** features 5 and 27, **(C)** features 4 and 24, and **(D)** features 14 and 23.

Again, a 2-dimensional feature study was performed. Two 2-dimensional datasets, Figures 5A,B, were created by randomly selecting two features from the breast cancer dataset. In addition, a dataset Figure 5C, with the two highest ranking features and a dataset, Figure 5D with the third and fourth highest ranking features were created by using the ranks of a chi-square test. The datasets are shown in Figure 5. The accuracy measurements are given in Table 8 and F1-scores in the Appendix in Table C4. A grid search with the grid {−5, −4, …, 4, 5} was also executed to obtain a baseline. Therefore, Algorithm 5 was used as an optimization procedure for the training method in Algorithms 3, 4 to create predictions.

**Table 8:** Improvement δ in accuracy for different optimization settings on 2-dimensional subsets of the breast cancer dataset.

	**Linear [%]**	**KNN [*%*]**	**Bayesian [%]**	**SVC [%]**	**NN [%]**
Base accuracy	75.610	79.615	81.622	82.678	83.66
Diagonal	0.664	–0.177	0.843	0.456	0.283
Spherical	6.258	–0.105	1.161	0.456	–0.138
MLE	0.107	0.176	1.197	–0.211	–0.034
2D grid search (δ)	7.064	0.176	1.019	0.736	–0.175
Iterative grid search (δ)	6.153	0.034	0.951	0.490	–0.245
Shift (δ)	6.293	0.034	0.845	0.490	–0.175
Shuffle (δ)	6.187	0.034	0.635	0.455	–0.140
Finer (δ)	6.222	0.068	0.635	0.490	–0.105
Combined 1 (δ)	5.871	0.246	0.705	0.490	–0.070
Combined 2 (δ)	6.713	–0.037	0.739	0.597	0.001
Features 5 and 27					
Base accuracy	76.007	84.538	84.677	85.736	86.754
Diagonal	2.421	–0.070	0.987	0.383	–0.352
Spherical	8.393	0.142	0.107	0.489	–0.175
MLE	3.261	–0.070	0.140	0.842	–1.266
2D grid search (δ)	8.566	0.072	–0.034	0.138	–0.175
Iterative grid search (δ)	8.919	0.071	–0.034	0.383	–0.317
Shift (δ)	8.568	0.071	–0.174	0.348	–0.598
Shuffle (δ)	8.919	0.071	–0.034	0.383	–0.317
Finer (δ)	9.200	0.036	–0.174	0.489	–0.457
Combined 1 (δ)	9.235	0.142	–0.315	0.419	–0.527
Combined 2 (δ)	9.060	0.177	0.177	0.419	–0.493
Features 4 and 24					
Base accuracy	90.468	92.864	90.893	92.439	92.829
Diagonal	0.148	–0.106	–0.352	–0.281	0.142
Spherical	1.484	0.211	–0.246	–0.211	0.281
MLE	–0.207	0.000	–2.494	–0.174	0.211
2D grid search (δ)	1.553	–0.211	0.140	–0.281	0.212
Iterative grid search (δ)	1.694	–0.140	–0.246	–0.175	0.177
Shift (δ)	1.694	–0.105	–0.387	–0.316	0.212
Shuffle (δ)	1.694	–0.140	–0.246	–0.175	0.177
Finer (δ)	1.519	–0.246	–0.246	–0.175	0.387
Combined 1 (δ)	1.519	–0.070	–0.246	–0.211	0.352
Combined 2 (δ)	1.729	–0.387	–0.106	–0.281	0.211
Features 14 and 23					
Base accuracy	88.435	90.439	90.966	92.407	92.057
Diagonal	0.695	–0.247	0.632	–0.421	–0.702
Spherical	3.339	–0.316	–0.071	–0.035	–0.597
MLE	–0.423	–0.037	0.738	–0.595	–0.352
2D grid search (δ)	3.620	–0.315	0.385	0.528	–0.773
Iterative grid search (δ)	3.409	–0.386	0.349	–0.350	–0.387
Shift (δ)	3.586	–0.316	0.384	–0.386	–0.492
Shuffle (δ)	3.409	–0.386	0.349	–0.350	–0.387
Finer (δ)	3.516	–0.352	0.384	–0.350	–0.387
Combined 1 (δ)	3.586	–0.422	0.419	–0.385	–0.598
Combined 2 (δ)	2.919	–0.387	0.385	–0.281	–0.457

*The highest improvement was achieved by the linear classifier for features [5, 27] with 9.235%. In 13 out of the 20 feature classification cases (4 tests × 5 classifiers), the iterative optimization improved the overall accuracy of the base classification models. Moreover, they achieved higher accuracy compared to the 2D grid search in 12 out of 20 cases, 16 out of 20 cases compared to Diagonal optimization, 15 out of 20 cases compared to Spherical optimization and 13 out of 20 cases compared to MLE. The best hyperparameter choice was dependent on the data as well as the classifier*.

The iterative method resulted in an improvement of the base accuracy in 13 out of the 20 cases for at least one hyperparameter setting. The highest loss of the best hyperparameter setting was −0.387%. This was realized by the neural network for features [14, 23]. In contrast, the highest gain of 9.235% was achieved by the linear classifier for features [5, 27]. Compared to a grid search, the iterative method was able to achieve the same or better performance in 13 out of 20 cases for at least one hyperparameter setting. The linear classifier always benefited from the proposed optimization. The Bayesian classifier also achieved consistent improvements, except for features [4, 24]. The KNN classifier performance improved only in half of the cases (features [2, 6] and [5, 27]), as did that of the neural network (features [4, 24] and [2, 6]). As already observed in the experiment with the sonar dataset, *Shift*, and *Finer* both increased and decreased the performance compared to the *Iterative grid search* optimization. The influence varied from classifier to classifier and from dataset to dataset. For example, for features [2, 6] the linear classifier did benefit from the *Shift*, but the Bayesian classifier did not. In contrast, the linear classifier for features [5, 27] experienced a decrease in accuracy. *Shuffle* did not further influence the performance of the *Iterative grid search* method. *Combined 1* and *Combined 2* had varying influences depending on the classifier and the dataset. Compared to the proposed optimization, *Diagonal* optimization was worse in 16 out of 20 cases, *Spherical* optimization in 15 out of 20 cases, and *MLE* in 13 out of 20 cases. The F1-scores are given in the Appendix in Table C4 and comparable results were measured. The proposed optimization achieved a better score in 10 cases compared to the base accuracy, in 12 cases compared to *2D grid search*, in 14 cases *Diagonal* optimization, in 15 cases compared to *Spherical* optimization, and in 13 cases compared to the *MLE*.

## Discussion

With grid exploration, we have shown that the Box-Cox transformation is able to consistently improve the accuracy. This behavior was also observed by Bicego and Baldo (2016). According to their results, we have also demonstrated that optimization depends on the classifier. Further, we observed that *full* optimization leads to higher improvements. Therefore, suitable optimization is introduced. This method is further improved to be able to handle different problems during optimization. From two real-world datasets, we demonstrated that the proposed procedure is able to achieve improvements in accuracy and F1-score. Furthermore, we have shown that this optimization is superior to grid searches, *diagonal, spherical*, and *MLE* optimization in the majority of cases. We suspect that the iterative procedure introduces some implicit regularization. Grid search is likely to overfit the training data, whereas the iterative method might not be able to find a global solution on the training set and hence suffers less from overfitting. The proposed optimization also scales linearly with the ability to support finer grids. Real-world dataset studies have shown that the hyperparameter setting is dependent on the data itself and the classifier. Restarting the optimization with multiple starting points and refining the grid influenced the results. However, shuffling the optimization order did not have a meaningful impact.

The Box-Cox transformation is data-dependent. Hence, the optimal choice of λ varies, and we recommend using an appropriate optimization method. We have demonstrated that the optimization method should take the classifier into account. However, non-classifier-dependent optimization methods like MLE might also perform well. Therefore, the best approach to obtain the best Box-Cox transformation is to evaluate different optimization procedures and compare the results.

## Conclusion

The impact of the Box-Cox transformation in classification tasks was examined. We extended the optimization of the parameters to a full dataset dependent problem and showed that this generalization improved the performance. An optimization procedure was proposed, successfully tested, and improvements up to 12% could be achieved.

In future work, an extensive application of the method to various datasets should be used to test the ability of the optimization. The influence of the hyperparameters should also be analyzed. Furthermore, the optimization could be improved by, for instance, replacing the 1-dimensional grid search with another 1-dimensional optimization. Although the Box-Cox transformation has been shown to increase the accuracy of a base classifier, it remains unclear whether it is also able to push the results of a classifier beyond state-of-the-art performance. Finally, the framework is designed with a general train-predict functionality that is often used in machine learning. Therefore, our method could also be applied to other tasks such as regression.

## Data Availability Statement

The developed code and data are publicly available via the following link: github.com/Luca-Blum/Box-Cox-for-machine-learning.

## Author Contributions

ME designed and led the study. LB, ME, and CM conceived the study. All authors approved final manuscript.

## Conflict of Interest

The authors declare that the research was conducted in the absence of any commercial or financial relationships that could be construed as a potential conflict of interest.

## Publisher's Note

All claims expressed in this article are solely those of the authors and do not necessarily represent those of their affiliated organizations, or those of the publisher, the editors and the reviewers. Any product that may be evaluated in this article, or claim that may be made by its manufacturer, is not guaranteed or endorsed by the publisher.
