# Supplementary material for: Impact of Box-Cox Transformation on Machine-Learning Algorithms
Source: Front Artif Intell. 2022 Apr 7;5:877569. doi: 10.3389/frai.2022.877569 (PMC9071306; doi:10.3389/frai.2022.877569)
Supplement: Supplementary file 1 [file Data_Sheet_1.pdf]

Appendix A Additional grid exploration heatmaps for artificial datasets

Following the heatmaps of the grid exploration for the *Gaussian quantiles* 1a, *interleaving half circles* 1b and *isotropic Gaussian blobs* 1c.

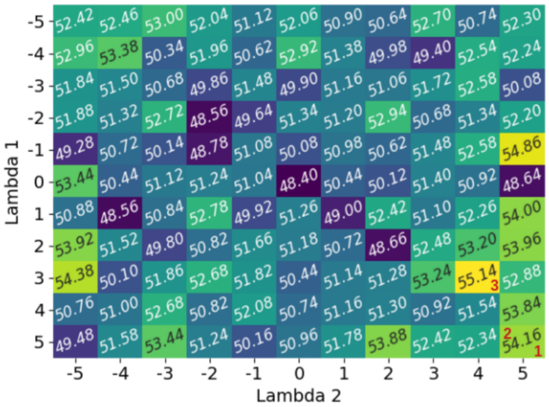

(a) Linear classifier

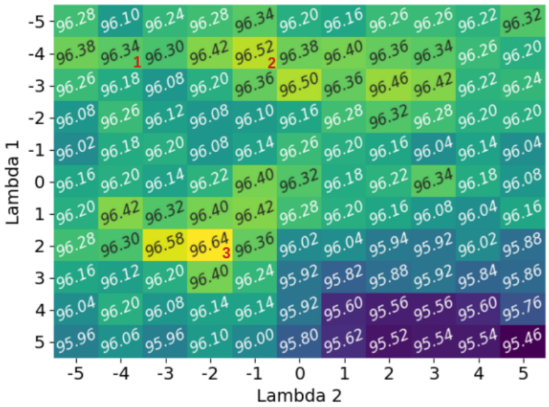

(b) KNN classifier

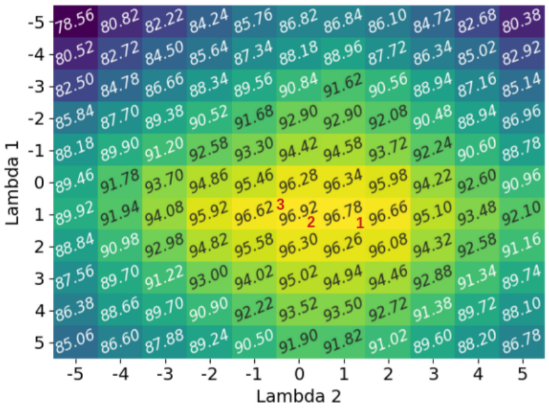

(c) Bayesian classifier

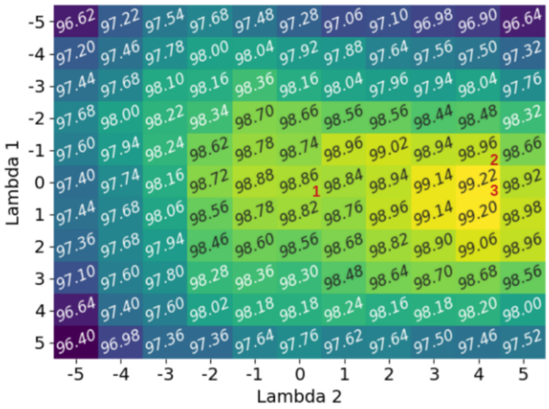

(d) SVC classifier

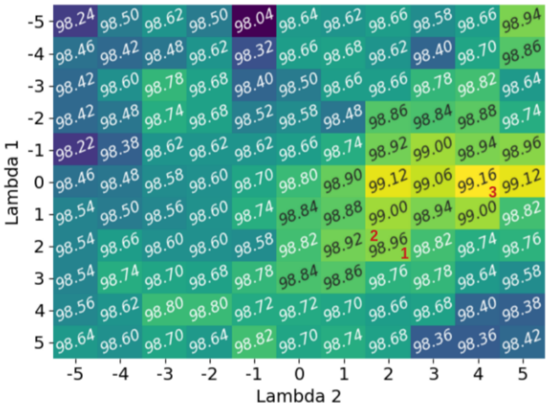

(e) NN classifier

Figure A1: Accuracy heatmaps generated by Algorithm 1 for Gaussian quantiles. The numbers 1, 2 and 3 correspond to the optimal solution for the *spherical*, *diagonal*, and *full* optimization. If there are multiple solutions then only one possibility is shown. It was observed that the optimal parameter choice for the Box-Cox transformation depends on the classifier. The heatmaps showed multiple local maxima and *full* optimization led to the best optimization result.

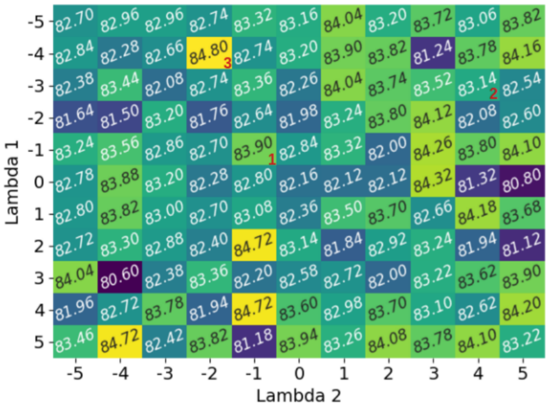

(a) Linear classifies

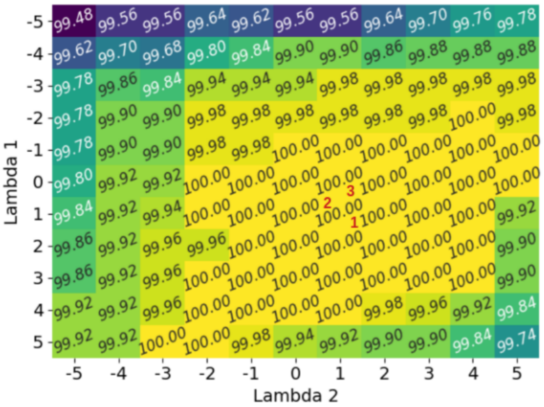

(b) KNN classifier

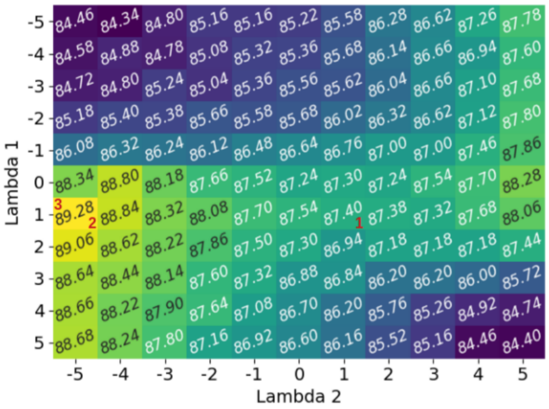

(c) Bayesian classifier

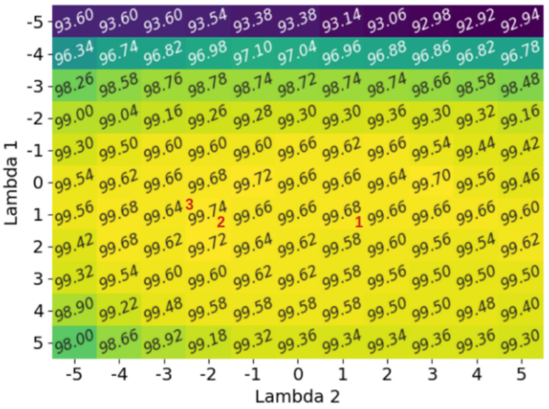

(d) SVC classifier

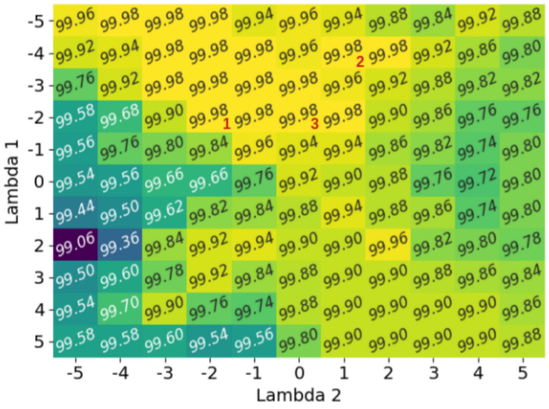

(e) NN classifier

Figure A2: Accuracy heatmaps generated by Algorithm 1 for interleaving half circles. The numbers 1, 2 and 3 correspond to the optimal solution for the *spherical*, *diagonal*, and *full* optimization. If there are multiple solutions then only one possibility is shown. It was observed that the optimal parameter choice for the Box-Cox transformation depends on the classifier. The heatmaps showed multiple local maxima and *full* optimization led to the best optimization result.

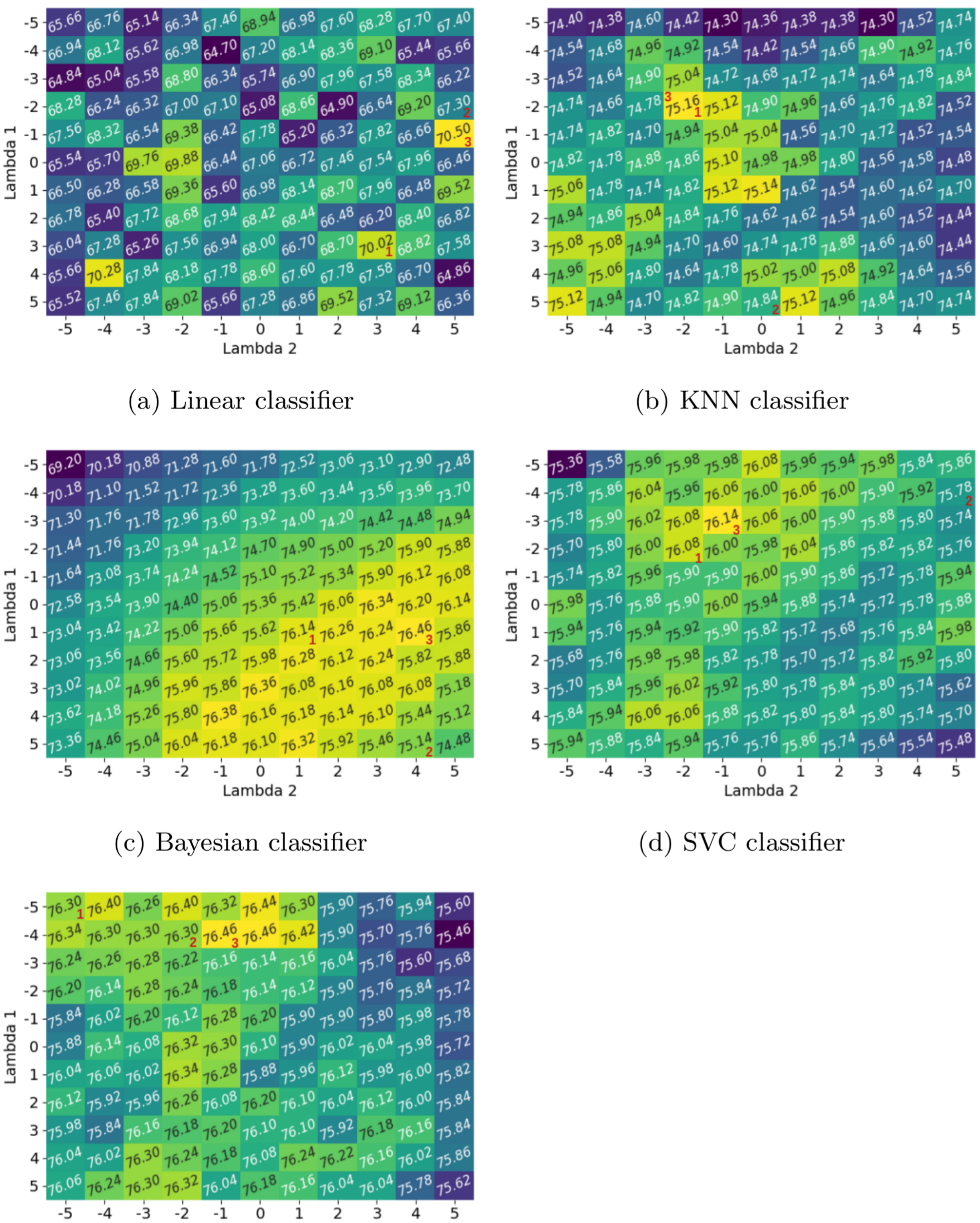

(a) Linear classifier

(b) KNN classifier

(c) Bayesian classifier

(d) SVC classifier

(e) NN classifier

Figure A3: Accuracy heatmaps generated by Algorithm 1 for isotropic Gaussian blobs. The numbers 1, 2 and 3 correspond to the optimal solution for the *spherical*, *diagonal*, and *full* optimization. If there are multiple solutions then only one possibility is shown. It was observed that the optimal parameter choice for the Box-Cox transformation depends on the classifier. The heatmaps showed multiple local maxima and *full* optimization led to the best optimization result.

Appendix B F1-scores for sonar dataset

Table B1: Improvement δ in F1-score for different iterative optimization settings in the sonar dataset. The proposed optimization achieved a consistent improvement except for the neural network. The different hyperparameter settings had a varying influence on the classifiers defined in Table 4. *Combined 2* improved the linear, KNN, and Bayesian classifier, whereas *Shift* already delivered the best performance for SVC. Additionally, the proposed optimization achieved higher improvements than *Diagonal*, *Spherical*, and *MLE* except for the neural network.

	Linear [%]	KNN [%]	Bayesian [%]	SVC [%]	NN [%]
Base accuracy	71.796	77.124	69.761	81.841	82.151
Diagonal	1.285	0.109	-0.342	-0.454	-0.929
Spherical	1.432	0.241	0.698	-0.322	-1.937
MLE	0.558	-0.675	1.641	2.513	-0.176
Iterative grid search (δ)	1.171	2.123	4.020	2.915	-0.208
Shift (δ)	0.988	2.208	4.020	2.999	-0.208
Shuffle (δ)	1.171	2.123	3.953	2.915	-0.208
Finer (δ)	0.867	2.123	3.959	2.915	-0.208
Combined 1 (δ)	0.576	2.001	3.585	2.771	-0.208
Combined 2 (δ)	4.095	4.153	4.366	2.550	-0.352

Table B2: Improvement δ in F1-score for different iterative optimization settings on 2-dimensional subsets of the sonar dataset. In 16 out of the 20 feature classification cases (4 tests $\times$ 5 classifiers), an improvement was achieved compared to the base accuracy. In 13 out of the 20 cases, iterative optimization was better than a 2D grid search, in 13 out of 20 cases better than *Diagonal* optimization, in 16 out of 20 cases better than *Spherical* optimization, and in 12 out of 20 cases better than *MLE*. The influence of the hyperparameter settings is data and classifier dependent.

	Linear [%]	KNN [%]	Bayesian [%]	SVC [%]	NN [%]
		Features 8 and 41			
Base accuracy	37.734	48.991	57.019	53.41	54.141
Diagonal	1.908	-0.208	-8.924	-0.971	0.019

To be continued

Table B2 (continued)

	Linear [%]	KNN [%]	Bayesian [%]	SVC [%]	NN [%]
Spherical	15.178	0.562	-5.978	-0.134	-0.164
MLE	3.470	-1.054	-4.630	0.649	-0.373
2D grid search (δ)	16.257	-1.973	-5.089	-0.758	-0.672
Iterative grid search (δ)	17.481	-3.340	-4.474	-0.900	0.837
Shift (δ)	18.244	-3.007	-4.341	-1.044	-0.079
Shuffle (δ)	17.481	-3.340	-4.474	-0.900	0.837
Finer (δ)	18.731	-3.304	-3.896	-0.974	0.077
Combined 1 (δ)	18.758	-4.284	-3.697	0.316	0.094
Combined 2 (δ)	17.090	-2.864	-2.690	-0.211	0.010
Features 2 and 48					
Base accuracy	49.224	61.746	66.895	63.936	62.442
Diagonal	6.324	-0.095	-2.045	-1.909	-2.883
Spherical	14.404	-0.193	-2.954	-1.469	-0.848
MLE	-9.204	1.083	-0.628	-2.108	-1.899
2D grid search (δ)	14.139	-0.308	-2.058	-1.626	-0.841
Iterative grid search (δ)	14.979	-0.464	-2.397	-2.652	-0.785
Shift (δ)	14.788	-0.380	-2.397	-2.652	-0.478
Shuffle (δ)	14.979	-0.464	-2.397	-2.652	-0.785
Finer (δ)	14.963	0.301	-2.804	-2.448	-1.045
Combined 1 (δ)	13.616	0.016	-2.737	-2.644	-0.906
Combined 2 (δ)	14.085	-1.009	-2.603	-2.655	-1.252
Features 11 and 45					
Base accuracy	58.02	67.123	67.596	66.625	66.698
Diagonal	3.012	-0.431	-0.232	-0.940	0.849
Spherical	7.037	-0.499	-1.947	-0.078	-0.889
MLE	2.467	-1.759	-1.464	-0.542	0.515
2D grid search (δ)	9.705	-0.560	-0.251	0.093	-0.628
Iterative grid search (δ)	9.136	-0.866	0.586	0.054	-1.305
Shift (δ)	9.410	-0.835	0.510	-0.108	-0.781
Shuffle (δ)	9.136	-0.866	0.586	0.054	-1.305
Finer (δ)	9.370	-0.579	0.627	0.149	-1.048
Combined 1 (δ)	9.392	-0.689	0.627	0.185	-0.932

To be continued

Table B2 (continued)

	Linear [%]	KNN [%]	Bayesian [%]	SVC [%]	NN [%]
Combined 2 (δ)	9.952	-0.929	0.950	0.325	-0.763
			Features 12 and 36		
Base accuracy	53.077	66.737	68.198	63.974	65.989
Diagonal	4.703	-1.214	-4.076	1.954	0.494
Spherical	12.271	-1.602	-2.746	0.798	-0.276
MLE	6.340	-2.009	-1.249	0.963	0.504
2D grid search (δ)	13.975	0.356	-4.443	1.556	-0.660
Iterative grid search (δ)	12.999	1.840	-4.137	0.602	0.207
Shift (δ)	12.936	1.840	-4.137	0.767	-0.168
Shuffle (δ)	12.999	1.840	-4.137	0.602	0.207
Finer (δ)	13.346	1.941	-4.647	0.475	-0.228
Combined 1 (δ)	13.546	2.081	-4.778	0.951	0.496
Combined 2 (δ)	14.106	1.992	-4.368	0.313	0.825

Appendix C F1-score for breast cancer dataset

Table C3: Improvement δ in F1-score for different iterative optimization settings in the breast cancer dataset. The proposed optimization achieved a consistent improvement except for the neural network. The different hyperparameter settings had a varying influence on the classifiers defined in Table 4. *Combined 2* improved the linear, KNN, and Bayesian classifier, whereas *Shift* already delivered the best performance for SVC. *Diagonal* optimization was better than the proposed optimization for the SVC and NN. *Spherical* optimization was better for the linear and Bayesian classifiers. *MLE* optimization was better for the Bayesian classifier, SVC, and NN.

	Linear [%]	KNN [%]	Bayesian [%]	SVC [%]	NN [%]
Base accuracy	97.212	97.543	94.693	98.046	98.507
Diagonal	0.254	-0.126	0.987	0.283	-0.205
Spherical	0.334	-0.039	1.162	0.148	-0.998
MLE	-0.146	0.192	1.171	0.205	-0.224
Iterative grid search (δ)	0.142	0.159	1.071	0.176	-0.482
Shift (δ)	0.138	0.021	0.976	0.091	-0.635

To be continued

Table C3 (continued)

	Linear [%]	KNN [%]	Bayesian [%]	SVC [%]	NN [%]
Shuffle (δ)	0.142	0.159	1.044	0.176	-0.482
Finer (δ)	0.255	0.159	1.071	0.176	-0.482
Combined 1 (δ)	0.056	0.213	1.051	0.176	-0.457
Combined 2 (δ)	0.166	0.081	1.136	0.176	-0.540

Table C4: Improvement δ in F1-score for different iterative optimization settings on 2-dimensional subsets of the breast cancer dataset. In 10 out of the 20 feature classification cases (4 tests $\times$ 5 classifiers), an improvement was achieved compared to the base accuracy. In 12 out of the 20 cases, iterative optimization was better than a 2D grid search, in 14 cases better than *Diagonal* optimization, in 15 cases better than *Spherical* optimization, and in 13 cases better than *MLE*. The influence of the hyperparameter settings is data and classifier dependent.

	Linear [%]	KNN [%]	Bayesian [%]	SVC [%]	NN [%]
Features 2 and 6					
Base accuracy	78.015	83.918	86.215	86.393	87.270
Diagonal	2.525	-0.101	-0.186	0.321	0.189
Spherical	7.596	-0.086	-0.018	0.342	-0.178
MLE	2.320	0.301	-0.019	-0.207	-0.044
2D grid search (δ)	8.549	0.118	0.116	0.568	-0.206
Iterative grid search (δ)	7.661	0.019	-0.384	0.366	-0.145
Shift (δ)	7.706	0.020	-0.004	0.386	-0.279
Shuffle (δ)	7.661	0.019	-0.384	0.366	-0.145
Finer (δ)	7.885	0.024	-0.337	0.397	-0.132
Combined 1 (δ)	7.603	0.144	-0.277	0.397	-0.112
Combined 2 (δ)	8.284	-0.058	-0.200	0.456	-0.017
Features 5 and 27					
Base accuracy	79.495	87.497	88.429	88.753	89.278
Diagonal	2.520	-0.033	0.187	0.203	-0.211
Spherical	7.835	0.161	-0.883	0.328	-0.131
MLE	3.389	-0.049	-0.664	0.606	-0.875

To be continued

Table C4 (continued)

	Linear [%]	KNN [%]	Bayesian [%]	SVC [%]	NN [%]
2D grid search (δ)	8.072	0.053	-0.950	0.017	-0.053
Iterative grid search (δ)	8.326	0.038	-0.992	0.216	-0.173
Shift (δ)	8.024	0.038	-0.973	0.186	-0.409
Shuffle (δ)	8.326	0.038	-0.992	0.216	-0.173
Finer (δ)	8.567	0.011	-1.095	0.290	-0.306
Combined 1 (δ)	8.516	0.095	-1.162	0.230	-0.364
Combined 2 (δ)	8.555	0.155	-0.791	0.203	-0.334
Features 4 and 24					
Base accuracy	92.386	94.402	93.083	94.207	94.373
Diagonal	-0.030	-0.034	-0.304	-0.223	0.119
Spherical	1.326	0.172	-0.201	-0.172	0.200
MLE	-0.549	0.000	-2.212	-0.180	0.151
2D grid search (δ)	1.371	-0.142	0.117	-0.219	0.151
Iterative grid search (δ)	1.474	-0.104	-0.188	-0.158	0.125
Shift (δ)	1.466	-0.079	-0.280	-0.257	0.156
Shuffle (δ)	1.474	-0.104	-0.188	-0.158	0.125
Finer (δ)	1.326	-0.180	-0.188	-0.153	0.288
Combined 1 (δ)	1.315	-0.042	-0.188	-0.178	0.259
Combined 2 (δ)	1.489	-0.286	-0.077	-0.228	0.144
Features 14 and 23					
Base accuracy	90.060	92.491	93.192	94.114	93.772
Diagonal	0.798	-0.207	0.408	-0.322	-0.569
Spherical	3.553	-0.253	-0.101	-0.015	-0.476
MLE	0.515	-0.043	0.378	-0.515	-0.262
2D grid search (δ)	3.826	-0.251	0.212	0.445	-0.608
Iterative grid search (δ)	3.586	-0.308	0.184	-0.281	-0.293
Shift (δ)	3.777	-0.250	0.208	-0.308	-0.375
Shuffle (δ)	3.586	-0.308	0.184	-0.281	-0.293
Finer (δ)	3.723	-0.272	0.202	-0.281	-0.293
Combined 1 (δ)	3.775	-0.322	0.235	-0.309	-0.460
Combined 2 (δ)	3.142	-0.301	0.207	-0.217	-0.350
